# Grape dataset: A dataset for disease prediction and classification for machine learning applications through environmental parameters

**DOI:** 10.1016/j.dib.2024.110546

**Published:** 2024-05-21

**Authors:** Apeksha Gawande, Swati Sherekar

**Affiliations:** Sant Gadge Baba University, SGBAU, Amravati, India

**Keywords:** Classification, Disease, Dataset, Machine learning, Powdery

## Abstract

Grapes are indeed a vital crop for both the fruit and wine industries globally. They are cultivated in numerous regions around the world and contribute significantly to the economy and culinary culture of many countries. By prioritizing disease detection and implementing proactive management strategies, growers can effectively safeguard their grape crops, optimize yields, and sustain the productivity and profitability of the global fruit and wine industry. We present the “Grape Disease Dataset” consisting of 10,000 records of classified environmental parameters sensed by sensors classified into three categories, temperature, humidity, and leaf wetness. The dataset covers diseases such as powdery mildew, downy mildew, and bacterial leaf spot. Designed to meet the needs of researchers and practitioners who wish to develop machine learning algorithms for disease detection. Some of the most common diseases affecting grapes include fungal infections such as powdery mildew, downy mildew, bacterial leaf spot and bunch rot. These diseases can affect different parts of the grapevine, including leaves, shoots, and clusters, ultimately leading to reduced photosynthesis, defoliation, and loss of fruit quality. Additionally, bacterial diseases like Pierce's disease and viral diseases like grapevine leaf roll disease pose serious threats to grape cultivation. Managing these diseases requires a combination of preventive measures, cultural practices, and, in some cases, the use of fungicides, bactericides, or other chemical treatments. Additionally, advancements in disease-resistant grape varieties and sustainable farming practices are being explored to mitigate the impact of diseases on grape production. Overall, effective disease management strategies are crucial for maintaining the health and productivity of grapevines, ensuring a stable supply of grapes for both fresh consumption and wine making, and sustaining the global fruit and wine industry. In order to improve the accuracy and efficiency of automated grape disease identification systems, various machine learning techniques can be applied to the dataset, including feature extraction and pattern recognition.

Specifications Table

**Table:** 

Subject	Computer Science, Applied Machine Learning, Agriculture
Specific subject area	Agronomy & Crop Science
Type of data Type of format	Figure Analyzed
Data collection	Data can be collected remotely or on field. Actual results can be taken from the field at real time at regular interval. The data collection process involved capturing environmental parameters like temperature, humidity and leaf wetness under diverse scenarios. Temperature and Leaf Wetness Sensor are needed to measure weather parameters and managed by NodeMCU. Data set were analyzed to predict the instances of diseases Downey Mildew, Powdery Mildew and Bacterial Leaf Spot using the algorithm.
Data source location	Malode Grape Farm, Eklahare, Nashik, Maharashtra, India, 422105
Data accessibility	Repository name: Grape Disease Dataset Data identification number: 10.17632/94j4ws2325.1 Direct URL to data: https://data.mendeley.com/datasets/94j4ws2325/1
Related research article	

## 1 Value of the Data

• *Research and Analysis:* Researchers can analyze the dataset to identify correlations between environmental factors and disease occurrence. By studying how variables such as temperature, humidity, and leaf wetness relate to the presence or severity of diseases like powdery mildew, downy mildew, and bacterial leaf spot, they can gain insights into the underlying mechanisms of disease development.
• *Model Development:* The dataset can be used to develop predictive models for disease occurrence. Machine learning algorithms can be trained on the data to predict the likelihood of disease outbreaks based on environmental conditions. These models can aid in early detection and proactive management strategies.
• *Improving Disease Detection Accuracy:* By incorporating environmental parameters into disease detection algorithms, accuracy can be enhanced. Timely detection of diseases which may not exhibit visible symptoms until later stages, can be improved by considering environmental cues that precede symptom expression.
• *Control and Management Strategies:* Understanding the relationship between environmental conditions and disease occurrence enables more targeted control and management strategies. Growers can implement preventive measures such as adjusting irrigation schedules or applying fungicides based on real-time environmental data, reducing the risk of disease outbreaks.
• *Mitigating Disease Impact:* By improving disease detection accuracy and implementing effective management strategies, the impact of grape diseases on yield and quality can be mitigated. This benefits both growers and the wider fruit and wine industry by ensuring a more stable and reliable supply of grapes.
• *Validation and Interpretation:* Validate the trained models using the validation set and interpret the results to understand which environmental factors are most influential in grape disease prediction and classification.

The dataset covering environmental parameters and grape diseases provides a valuable tool for research, modeling, and decision-making aimed at better understanding, detecting, and managing grape diseases.

## 2 Background

If any plant is suffering from any disease means it restricts it to give its best. Automatically there is a loss of production due to these diseases. Farmers usually do manual inspection and spray pesticides. Any error during the diagnosis of disease may lead to wrong controlling and excess use of pesticides. We need to find a solution to predict these diseases at early stage. There is a direct relationship between weather and crop diseases as well as weather monitoring and crop diseases [1].

The goal of our system is to collect information on climatic factors like temperature and humidity which helps to monitor health of plant as well as growth of any disease on plant. It will assist farmer to predict disease at early stage. Automatically system help farmer to increase the production [2]. It will be also useful for development of grape plantation and improvement in quality of grapes by displaying information regarding weather conditions and choosing which fungicides to use. We have developed integrated sensor network having high level sensing methodology with low cost. This network assists in monitoring different climatic condition. This system help farmer to monitor entire vineyard for quality grapes.

## 3 Data Description

Creating a dataset for grape disease prediction and classification involving environmental parameters would require a combination of grape-related data and environmental variables [3]. The environment plays a crucial role in shaping crop growth and productivity. At all times, the environment affects a crop and influences its production. An IoT will be utilized for monitoring temperature, humidity, and leaf wetness, all of which influence grape quality and lifespan. By monitoring environmental conditions, growers can take proactive measures to protect their crops and improve overall yield [4]. Furthermore, a far reaching dataset takes into consideration the investigation of illness designs, natural elements, and potential moderation systems. By leveraging weather-related variables and sensor data, grapevine producers can create an efficient and automated disease detection system. This paper presents a self-created weather parameter database using sensors. The database consists of 10,000 records divided into 5 categories. Here, experiment has been carried out using our dataset to predict grape diseases on various machine learning algorithm.

**Fig. 1:**
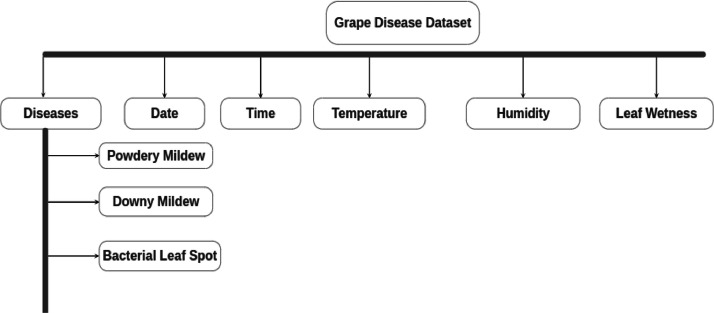
Structure of the Grape disease dataset.

**Fig. 2:**
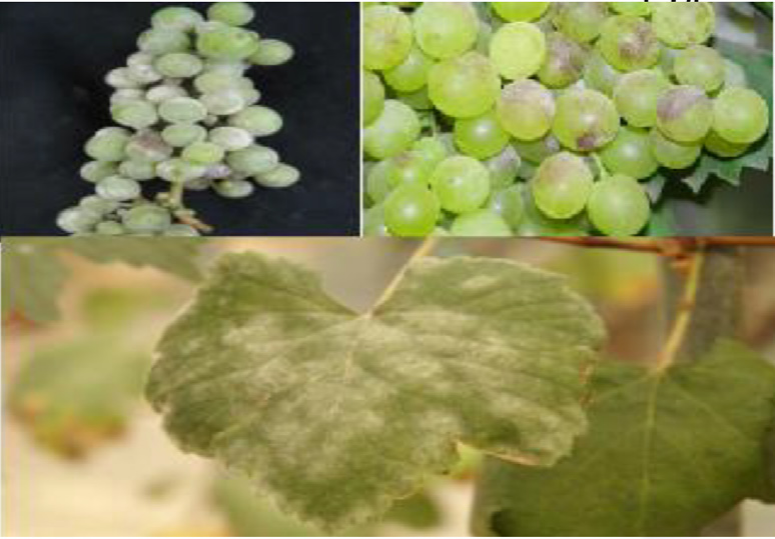
Powdery Mildew.

**Fig. 3:**
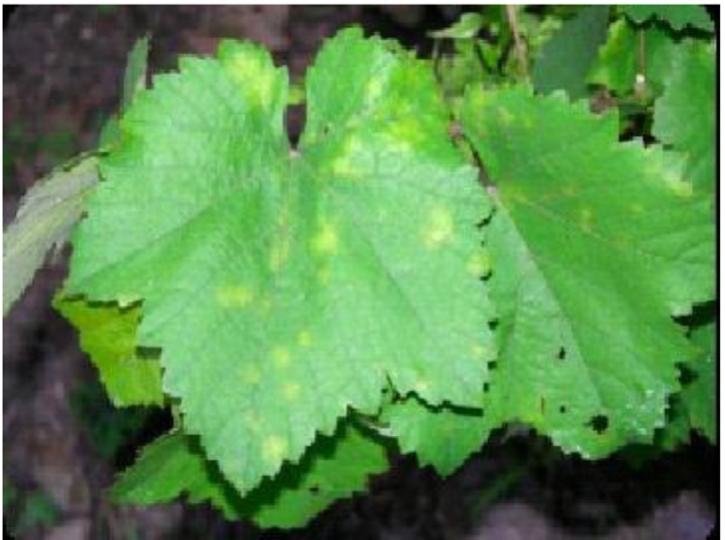
Downey Mildew.

**Fig. 4:**
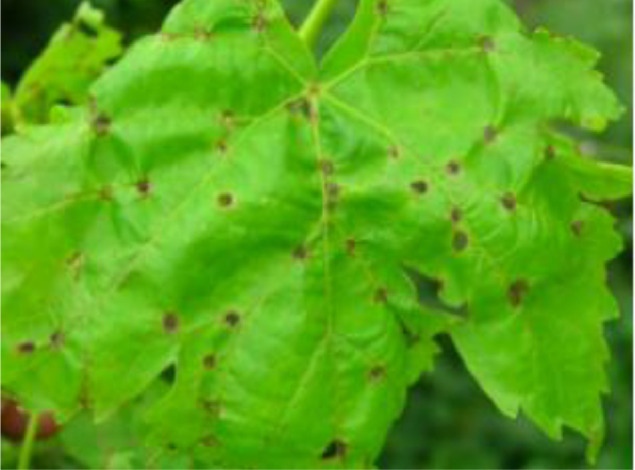
Bacterial leaf spot.

Data can be collected remotely or on field. Actual results can be taken from the field at real time after regular interval. Here, experiment has been carried out using our dataset to predict grape diseases on various machine learning algorithm. The dataset is categorized into 8 distinct classes, including 3 disease categories (Fig. 1). The disease categories cover a range of common grape diseases, such as powdery mildew (Fig. 2), downy mildew (Fig. 3), bacterial leaf spot (Fig. 4). Temperature and Leaf Wetness Sensor are needed to measure weather parameters and managed by NodeMCU. Data set were analyzed to predict the instances of diseases Downey Mildew, Powdery Mildew and Bacterial Leaf Spot using the algorithm.

## 4 Experimental Design, Materials and Methods

**Fig. 5:**
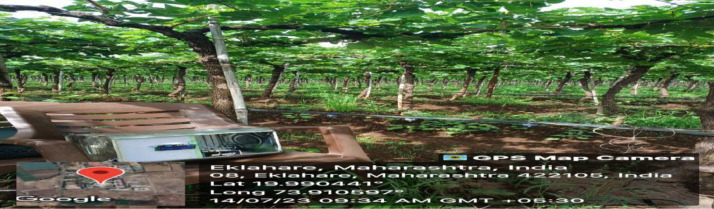
Experimental device setup.

The experimental setup we had deployed in the field and actual results captured from the field at real time. We had installed the system at Malode Grape Farm, Eklahare, Nashik, Maharashtra, India, 422105 shown in Fig. 5.

### 4.1 Specification of image acquisition system (Table 1)

**Table 1:** Specification of proposed system.

Sr. No	Component Description	Specification
1	Power Supply: Battery	40A, 14.8–16.8 V
2	Temperature Sensor	DHT11
3	Leaf wetness Sensor	-
4	GSM Module	SIM800L GSM/GPRS, 4 V
5	Node MCU	ESP8266, 16 Digital Pins, Analog −1 Pin
6	LCD Display	16×2

#### 4.1.1 Sensors

Temperature and Leaf Wetness Sensor are needed to measure weather parameters (Temperature, Humidity and Leaf wetness) and managed by NodeMCU.

##### Temperature sensor

Several advantages make DHT11 the most popular temperature and humidity module for Arduino and Raspberry Pi. Low power consumption and excellent long-term stability with accuracy can be obtained at a very low cost.

*Leaf wetness* Leaf wetness sensor is a vital device for monitoring and researching leaf wetness must be measured since it is used to track the likelihood that fungus or disease may spread on a plant.

#### 4.1.2 Node MCU

A widely used WiFi module ESP8266-12E forms the basis of NodeMCU, an open source development board with firmware. With NodeMCU, you can develop your own applications on an open-source development board using the widely used ESP8266-12E WiFi module.

It allows you to program the ESP8266 WiFi module with the simple and powerful LUA programming language or Arduino IDE.

#### 4.1.3 Data collection

Data can be collected remotely or on field. Actual results can be taken from the field at real time after regular interval.

#### 4.1.4 GSM module

Occurrence of diseases if any alerts messages are sent to the farmers. SIM800L GSM is taken for transmitting the message.

#### 4.1.5 LCD display

**Fig. 6:**
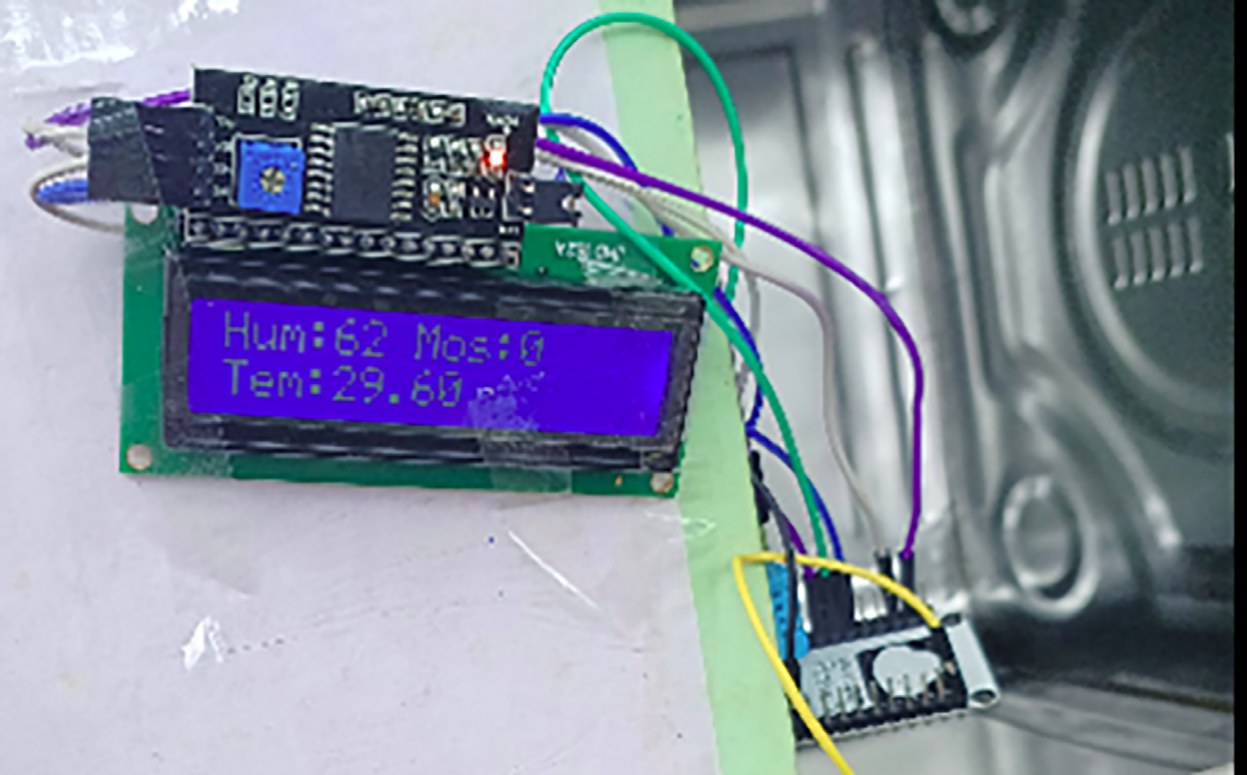
Temperature, Humidity and LW.

16×2 LCD display is selected for visualize purpose. Farmer can see name of the disease on LCD if occur or temperature, humidity and wetness at regular interval. Fig. 6 Showing weather parameters Temperature, Humidity and LW [3].

## 5 Economic Impact of Grape Disease in Different Regions

The prevalence and economic impact of grape diseases vary depending on factors such as climate, geography, grape varieties grown, agricultural practices, and disease management strategies. Powdery mildew is one of the most widespread and economically significant grape diseases worldwide [5]. It thrives in temperate and warm climates with moderate humidity. Severe infections can lead to reduced grape quality, yield loss, and increased production costs due to the need for fungicide applications and labor-intensive management practices.

Downy mildew is prevalent in regions with high humidity and frequent rainfall, such as cool and coastal areas. It can cause significant yield losses and affect grape quality, especially if not managed properly. Fungicide resistance is a growing concern in some regions.

Viral diseases can be found in grapevine-growing regions worldwide and are often spread by infected planting material and vectors such as mealybugs and nematodes. Viral infections can reduce vine vigor, delay ripening, and decrease fruit quality, leading to economic losses for growers. Prevention and control often involve using certified virus-free planting material and managing vector populations.

Understanding the specific challenges posed by grape diseases in different regions is essential for developing effective disease management strategies tailored to local conditions and practices. Additionally, ongoing research and extension efforts help growers stay informed about emerging threats and best practices for disease prevention and control.

## Limitations

*Limited Scope:* Temperature, humidity, and leaf wetness sensors can provide valuable data, but they might not capture all relevant factors influencing grape diseases. Other factors such as soil moisture, sunlight exposure, and air quality could also play significant roles but might not be adequately monitored by these sensors alone.

*Cost Considerations:* Implementing and maintaining an IoT system with multiple sensors can be costly, especially for small-scale vineyards. The initial investment in sensors, data infrastructure, and analytics tools must be weighed against the potential benefits in disease prediction and management.

## Complex Challenges

Grape disease management presents several complex challenges due to the diversity of pathogens, environmental factors, and the need for sustainable practices.

1. Pathogen Diversity - Grapes are susceptible to various diseases caused by fungi, bacteria, viruses, and nematodes. Each pathogen has its own lifecycle, environmental requirements, and management strategies, making it challenging to develop a comprehensive approach.
2. Environmental Factors - Weather conditions such as temperature, humidity, and rainfall can greatly influence disease development. For example, fungal diseases like powdery mildew thrive in warm, humid conditions, while downy mildew prefers cooler, wet weather. Climate change adds another layer of complexity by altering traditional disease patterns and increasing the frequency of extreme weather events.
3. Pesticide Resistance - Overreliance on chemical pesticides has led to the development of resistant strains of pathogens, making them less susceptible to treatment. This necessitates the development of integrated pest management (IPM) strategies that incorporate cultural, biological, and chemical control methods to minimize pesticide use and delayx resistance.
4. Cultural Practices - Pruning, trellising, canopy management, and irrigation practices can all impact disease development by affecting air circulation, sunlight exposure, and moisture levels within the vineyard. Implementing proper cultural practices requires knowledge of both grape physiology and disease epidemiology.
5. Sustainable Solutions - There is growing consumer demand for sustainably produced grapes, which requires minimizing chemical inputs, conserving water and soil resources, and promoting biodiversity in the vineyard. Sustainable disease management involves balancing economic, environmental, and social considerations while maintaining grape quality and yield.

## Advantages

1. High Resolution and Accuracy – Grape disease datasets through environmental parameters provide detailed and accurate information on temperature, humidity, rainfall, and other environmental variables at specific locations and time intervals. This high resolution allows growers to monitor microclimatic conditions within their vineyards accurately.
2. Real Time Monitoring - Grape disease datasets often offer real-time or near-real-time data updates, allowing growers to track weather conditions continuously throughout the growing season. This real-time monitoring enables timely decision-making and proactive disease management strategies.
3. Long-Term Historical Data - Grape disease datasets typically include historical weather data spanning several years or decades. This historical data allows growers to analyze long-term trends, identify recurring patterns, and assess the risk of disease outbreaks based on past weather conditions.
4. Integration with Disease model - Grape disease datasets can be integrated with predictive disease models to forecast disease risk and optimize disease management strategies. By combining weather data with disease epidemiology models, growers can anticipate disease development, schedule preventive treatments, and reduce reliance on reactive interventions.
5. Remote Access - Grape disease datasets are often accessible remotely through online platforms, mobile applications, or APIs (Application Programming Interfaces). This accessibility allows growers to access weather information from any location using various devices, facilitating timely decision-making and collaboration among stakeholders.
6. Cost Effectiveness - Compared to traditional methods of weather monitoring (e.g., on-site weather stations), Grape disease dataset offer a cost-effective solution for accessing comprehensive weather information. This dataset may come with data visualization and analysis tools that enable growers to interpret complex weather data easily.

Overall, Grape disease datasets through environmental parameters offer valuable insights and decision support tools for grape disease management, empowering growers to implement proactive and data-driven strategies to minimize disease risks and optimize vineyard health and productivity.

## Ethics Statement

Our study does not involve studies with animals or humans. Therefore, we confirm that our research strictly adheres to the guidelines for authors provided by Data in terms of ethical considerations.

## CRediT authorship contribution statement

**Apeksha Gawande:** Methodology, Data curation, Writing – original draft. **Swati Sherekar:** Conceptualization, Writing – review & editing.

## Data Availability

Grape Disease Dataset (Original data)[6], [7] (Mendeley Data).
